# Supplementary material for: Onecut Factors and Pou2f2 Regulate the Distribution of V2 Interneurons in the Mouse Developing Spinal Cord
Source: Front Cell Neurosci. 2019 Jun 5;13:184. doi: 10.3389/fncel.2019.00184 (PMC6561314; doi:10.3389/fncel.2019.00184)
Supplement: TABLE S1 — Microarray comparison of control and of Hnf6-/-;Oc2-/- spinal cords. The differential expression of selected candidates is shown as fold change in Hnf6-/-;Oc2-/- vs. control spinal cords. Pval, p-value; adjpval, adjusted p-value. [file Table_1.pdf]

Gene	Symbol	Fold change	pvalue	adjpvalue
Polyamine modulated factor 1 binding protein 1	Pmfbp1	0.28	1.50E-10	3.80E-06
Pappalysin 2	Pappa2	0.33	5.31E-05	0.134
NK6 homeobox 3	Nkx6-3	0.62	1.70E-05	0.072
Secreted frizzled-related sequence protein 5	Sfrp5	0.69	0.002	0.481
Synuclein, gamma	Sncg	0.70	1.65E-04	0.220
Sema domain, immunoglobulin domain (Ig), short basic domain, secreted, (semaphorin) 3D	Sema3d	0.70	0.076	0.681
NK6 homeobox 2	Nkx6-2	0.70	1.62E-03	0.481
Dorsal inhibitory axon guidance protein	Draxin	0.70	2.17E-03	0.486
Stathmin-like 4	Stmn4	0.71	1.14E-03	0.220
Cadherin 9	Cdh9	0.71	0.123	0.732
Tubulin polymerization-promoting protein family member 3	Tppp3	0.74	8.53E-04	0.471
Paired-like homeodomain transcription factor 2	Pitx2	0.75	2.167E-03	0.486
Slowmo homolog 1 (Drosophila)	Slmo1	1.29	2.24E-03	0.486
a disintegrin-like and metallopeptidase (reprolysin type) with thrombospondin type 1 motif, 3	Adamts3	1.47	7.02E-04	0.445
Cadherin 8	Cdh8	1.48	0.078	0.683
POU domain, class 2, transcription factor 2	Pou2f2	1.57	5.62E-04	0.399
Kallikrein 1-related peptidase b22	Klk1b22	3.20	1.34E-03	0.473
Lysine (K)-specific demethylase 5D	Kdm5d	3.46	0.046	0.668

Supplementary Table S1
